# Maternal and neonatal factors associated with child development in Ceará, Brazil: a population-based study

**DOI:** 10.1186/s12887-021-02623-1

**Published:** 2021-04-07

**Authors:** Hermano A. L. Rocha, Christopher R. Sudfeld, Álvaro J. M. Leite, Márcia M. T. Machado, Sabrina G. M. O. Rocha, Jocileide S. Campos, Anamaria C. e Silva, Luciano L. Correia

**Affiliations:** 1 Department of Global Health and Population, Harvard T. H. Chan School of Public Health, 677 Huntington Ave, Boston, MA 02115 USA; 2 Department of Maternal and Child Health, Federal University of Ceará, Fortaleza, CE Brazil; 3 Department of Community Health, Federal University of Ceará, Fortaleza, CE Brazil; 4 ISEC, Unichristus University Center, Fortaleza, CE Brazil

**Keywords:** Child development, Determinants, Low birth weight, Breastfeeding

## Abstract

**Background:**

The first 1000 days of life are a critical period when the foundations of child development and growth are established. Few studies in Latin America have examined the relationship of birth outcomes and neonatal care factors with development outcomes in young children. We aimed to assess the association between pregnancy and neonatal factors with children’s developmental scores in a cross-sectional, population-based study of children in Ceará, Brazil.

**Methods:**

Population-based, cross-sectional study of children aged 0–66 months (0–5.5 years) living in Ceará, Brazil. We examined the relationship of pregnancy (iron and folic acid supplementation, smoking and alcohol consumption) and neonatal (low birth weight (LBW) gestational age, neonatal care interventions, and breastfeeding in the first hour) factors with child development. Children’s development was assessed with the Ages and Stages Questionnaire (ASQ-BR). We used multivariate generalized linear models that accounted for clustering sampling to evaluate the relationship of pregnancy and neonatal factors with development domain scores.

**Findings:**

A total of 3566 children were enrolled. Among pregnancy factors, children whose mothers did not receive folic acid supplementation during pregnancy had lower fine motor and problem-solving scores (*p*-values< 0.05). As for neonatal factors, LBW was associated with 0.14 standard deviations (SD) lower (CI 95% -0.26, − 0.02) communication, 0.24 SD lower (95% CI: − 0.44, − 0.04) fine motor and 0.31 SD lower (CI 95% -0.45, − 0.16) problem-solving domain scores as compared to non-LBW children (*p* values < 0.05). In terms of care, newborns that required resuscitation, antibiotics for infection, or extended in-patient stay after birth had lower development scores in selected domains. Further, not initiating breastfeeding within the first hour after birth was associated with lower gross motor and person-social development scores (*p*-values < 0.05).

**Conclusion:**

Pregnancy and neonatal care factors were associated with later child development outcomes. Infants at increased risk of suboptimal development, like LBW or newborns requiring extended in-patient care, may represent groups to target for supplemental intervention. Further, early integrated interventions to prevent adverse pregnancy and newborn outcomes may improve child development outcomes.

## Background

It is estimated that over 250 million children under the age of 5 years in low- and middle-income countries (LMIC) do not reach their full developmental potential [1]. The first 1000 days of life, from conception to the second birthday, are critical for children’s development due to rapid brain development [2] and early child development is a determinant of later-life academic achievement and human capital outcomes [3–5].

Child development can be affected by a combination of socioeconomic, environmental, nutritional, and social factors during pregnancy and the first years of life [2, 6]. Studies in both high-income and LMIC have identified multiple factors associated poverty are associated with suboptimal development, low maternal level of schooling, suboptimal breastfeeding, and lack of responsive caregiving [6–8]. Further adverse birth outcomes, including low birth weight (LBW; < 2500 g) have been reported to be associated with suboptimal development outcomes. Additionally, developmental delays may be found in as many as 50% of children born with very low birth weight (VLBW; < 1500 g) [9–12]. The LBW prevalence in Brazil in 2015 was 10.1% [13].

Nevertheless, very few large, population-based studies have assessed the association of pregnancy and neonatal care factors with development outcomes in newborns, infants, toddlers, and preschoolers, particularly in the context of Latin American [14]. In addition, a recent systematic review determined that data on the association between delivery and neonatal characteristics with child development outcomes are lacking [15].

We assessed the association of pregnancy and neonatal factors with communication, gross-motor, fine-motor, problem-solving, and personal-social developmental scores.. These observational evidence are intended to inform populations at risk for suboptimal development and for design of interventions to improve development outcomes.

## Methods

### Study design and population

We analyzed data from the *Pesquisa de Saúde Materno Infantil no Ceará* (PESMIC, *Maternal and Child Health Survey in Ceará*) and full details about the parent study methods and the child development assessment can be found elsewhere [16, 17]. Briefly, the PESMIC is a population-based, cross-sectional study on maternal and child health carried out in preschool children, aged 0 to 72 months, living in the state of Ceará, in northeastern Brazil. Ceará is one of Brazil’s poorest states, with a population of 9 million inhabitants living in a semiarid climate. Fortaleza (2.3 million inhabitants) is the capital city and urban commercial center of Ceará. The study area also includes rural areas of the state, where subsistence farming is the dominant type of agricultural activity.

PESMIC surveys were conducted in 1987, 1990, 1994, 2001, 2007, and 2017 using the same methods. For this analysis, we used child development data from the 2017 PESMIC survey, conducted from August to November 2017. The PESMICs used cluster sampling, based on the Brazilian Institute of Geography and Statistics (IBGE) census tracts, and stratification between the state capital city of Fortaleza, and the rural areas. The 2017 PESMIC surveyed 160 randomly selected census tracts, including 3200 households. Once a census tract was defined and its corresponding map obtained, the location of the cluster of 20 houses to be investigated was determined as follows: the starting point of the cluster (the first home to be visited) was randomly selected utilizing ArcGIS® software, GIS Inc. Households were visited consecutively, in a counterclockwise spiral fashion. Shops and abandoned buildings were excluded and replaced, and in the case of absent families, up to three return visits were made to obtain data. In each household, information was obtained about all children through the mother’s or primary caregiver’s report (97.2% were mothers). All data were collected on paper forms and were double-entered using the software EpiInfo™ 2000 (used only for data entry).

### Child development assessment

Child development was assessed using the Ages and Stages Questionnaire version 3 [18], a screening tool for child development delays, which has been validated in Brazil (ASQ-BR) [19]. Child development was only assessed for participants aged 0 to 66 months since the ASQ has only been validated in this age group. Five child development domains were measured in the ASQ-BR: communication, broad motor coordination, fine motor coordination, problem-solving, and personal-social skills [18]. The interviewers were trained on the use of the ASQ-BR for 20 h by medical professionals. In terms of scoring, a child’s domain score was considered invalid and considered missing if more than two items were skipped. The ASQ was administered to mothers/caregivers by trained interviewers and by direct observation of the child. If one or two items in one area were skipped, we provided an adjusted score by calculating the average score for the completed items in that area and attributed the average score to the missed item [18]. Age-standardized scores were calculated, and adjustments were made for children aged less than 24 months and who were born preterm by subtracting the number of weeks of prematurity from the child’s chronological age and then using this number to determine the appropriate ASQ questionnaire to be administered [18].

### Exposures of interest

In Brazil, all children receive a child health booklet at birth, in which health professionals record health information about the antenatal care, delivery, vaccination, and child growth and development. We used these data to evaluate birth weight and gestational age at birth. Low birth weight was defined as children born weighing less than 2500 g. At birth, gestational age was collected from the child health booklet and is usually estimated from the first obstetric ultrasound. Preterm was defined as less than 37 completed weeks gestation. Delivery care factors were also recorded in the booklet.

Standardized questionnaires were administered to the mother or head of the household. Prenatal (including life habits variables), delivery, and birth data were reported by the mother and confirmed by the child health booklet. When maternal reports and the booklet data were divergent, the health booklet data were preferentially selected.

### Statistical analysis

We analyzed age- and sex-standardized ASQ-BR scores [20] for children aged 5 months or older. For children younger than 5 months, we used US ASQ standards due to the lack of Brazilian standardized scores for young infants under 5 months of age [21]. First, the descriptive statistics are presented, adjusting for clustering by design. We used generalized linear models to determine the association of pregnancy, delivery, and postnatal factors with the ASQ-BR domain scores. We present standardized mean differences (SMD) to compare effect sizes to other studies. We took a causal approach to the multivariate analyses, which were minimally adjusted for age, sex, income, and interviewer, then fully adjusted models including common causes (confounders) of the exposures of interest and development outcomes. To avoid adjusting for potential downstream mediators, pregnancy models did not adjust for birth outcome and postnatal factors. We also examined potential effect modification of the association of LBW with child development outcomes based on biological plausibility. We used pairwise deletion to account for missing data. All study data were analyzed using SPSS, Version 23 (SPSS Statistics for Windows, Version 23.0. IBM Inc).

### Ethical aspects

Written informed consent was obtained from the participating women. The caregivers also provided written permission for their child’s participation in the study, and consent for mothers who were adolescent minors was obtained from their parents or legal guardians. The Research Ethics Committee in Brazil approved the PESMICs survey under number 73516417.4.0000.5049.

## Results

The study included a cross-sectional population-based sample of 3566 children from 3200 households. A summary of the study population characteristics is presented in Table 1. The mean child age was 31.8 ± 23.1 months, and the sample was equally distributed between males and females. The mean maternal schooling attainment was 4.4 ± 2.8 years, and the mean income was R$1090.4 (~ 280 USD) ±1017.9 reais per household. Among children in the sample, 7.7% were born LBW and 10.4% were born preterm (< 37 weeks). Almost all women reported taking iron and folic acid supplements during the pregnancy, while 6% reported smoking or drinking during the pregnancy. A total of 525 children (14%) required extended in-hospital stay after birth, and 5% required antibiotics in the immediate postnatal period. A total of 79.2% of children were breastfed within the first hour of life.

**Table 1:** Characteristics of 3566 children from 3200 families assessed with the ASQ-3 at 0–72 months of age in Ceará, Brazil

Characteristics		Mean ± SD or N (%)^a^
**Child and social factors**		
Child Age		31.8 ± 23.1
Infant		789 (22.1)
Toddler		1363 (38.2)
Preschooler		1414 (39.7)
Male child		1786 (50.0%)
Maternal level of schooling in years		4.4 ± 2.8
Monthly household income in Brazilian Reais		1090.4 ± 1017.9
**Low birth weight (< 2500 g)**		**278 (7.7)**
**Pregnancy factors**		
Maternal iron supplementation during pregnancy		3317 (93.0%)
Folic acid supplementation during pregnancy		3320 (93.1)
Smoking during pregnancy		216 (6.0)
Alcohol consumption during pregnancy		207 (5.8)
**Birth and neonatal factors**		
Gestational age	Preterm (< 37)	147 (10.4)
	Full-Term	1270 (89.6)
Extended in-patient hospital stay after birth	NICU or Incubator	251 (7.0)
	Nursery	274 (7.7)
Need for resuscitation after birth		36 (1.0)
Need for antibiotics after birth		191 (5.3)
**Breastfeeding factors**		
Breastfeeding initiated within the 1st hour of life		2825 (79.2)
**Child development**		
ASQ-BR age-standardized scores		
Communication		52.2 ± 11.5
Gross motor		55.4 ± 9.3
Fine motor		49.7 ± 13.7
Problem-solving		50.7 ± 12.5
Personal-Social		50.1 ± 11.7

^a^Values are expressed as means ± S.D.s or n (%); *n* = 3566. ASQ-3. Ages and Stages Questionnaire version 3

The association between pregnancy factors, LBW and prematurity with child development outcomes is shown in Table 2. In a multivariate analysis, not reporting prenatal folic acid supplementation was associated with lower fine-motor and problem-solving domain scores (*p* values < 0.01). LBW was also associated with lower communication (standardized mean difference (SMD): -0.14; 95% CI -0.26, − 0.02), fine motor (SMD: -0.24;95% CI -0.44, − 0.04) and problem-solving (SMD: -0.31; 95% CI -0.45, − 0.16) domain scores (*p* values < 0.05), after adjustment for the child’s age, sex, income, pregnancy factors and gestational age.

**Table 2:** Association pregnancy factors, low birthweight and prematurity with child developmental outcomes

	N	Communication	*p*-value	Gross motor	*p*-value	Fine motor	*p*-value	Problem-solving	*p*-value	Personal-social	*p*-value
		Multivariate-adjusted standardized mean difference (95% CI)		Multivariate-adjusted standardized mean difference (95% CI)		Multivariate-adjusted standardized mean difference (95% CI)		Multivariate-adjusted standardized mean difference (95% CI)		Multivariate-adjusted standardized mean difference (95% CI)	
***Birth weight***^**a**^ (reference: ≥ 2500 g)											
< 2500 g	278	-0.14 (−0.26, − 0.02)	**0.02**	− 0.17 (− 0.35, 0.01)	0.06	− 0.24 (− 0.44, − 0.04)	**0.01**	− 0.31 (− 0.45, − 0.16)	**< 0.001**	− 0.06 (− 0.21, 0.08)	0.38
***Gestational age*** *(reference: Full-term)*											
Preterm (< 37 weeks)	147	− 0.18 (− 0.20, 0.16)	0.84	− 0.11 (− 0.32, 0.09)	0.27	0.05 (− 0.20, 0.30)	0.70	− 0.08 (− 0.31, 0.13)	0.44	0.15 (− 0,02, 0.34)	0.09
***Pregnancy Factors***^***b***^											
Iron supplementation during pregnancy (Reference: yes)											
No	183	0.02 (− 0.19,0.23)	0.85	0 (− 0.22, 0.22)	0.99	0.23 (− 0.09, 0.56)	0.16	0.23 (− 0.05, 0.52)	0.11	0.03 (− 0.19, 0.26)	0.77
Folic acid supplementation during pregnancy (Reference: yes)											
No	177	− 0.06 (− 0.24, 0.12)	0.52	− 0.15 (− 0.37, 0.07)	0.18	− 0.53 (− 0.87, − 0.19)	**0.002**	− 0.34 (− 0.59, − 0.09)	**0.008**	−0.04 (− 0.26, 0.17)	0.67
Smoking during pregnancy (Reference: no)											
Yes	216	− 0.05 (− 0.24, 0.12)	0.54	− 0.01 (− 0.24, 0.22)	0.92	− 0.2 (− 0.42, 0.02)	0.07	− 0.22 (− 0.49, 0.03)	0.09	− 0.09 (− 0.27, 0.08)	0.29
Self-reported alcohol consumption during pregnancy (Reference: no)											
Yes	207	− 0.17 (− 0.34, 0)	0.05	0.03 (− 0.13, 0.21)	0.67	− 0.19 (− 0.4, 0.02)	0.08	− 0.11 (− 0.31, 0.07)	0.24	0.04 (− 0.12, 0.2)	0.62

^a^Adjusted for age, sex, interviewer, maternal level of schooling, permanent income, ferrous sulfate, folic acid, smoking, drinking, low birth weight, gestational age and twin birth

^b^Adjusted for age, sex, interviewer, maternal level of schooling, permanent income

The relationship of neonatal care factors with development outcomes is shown in Table 3. Extended in-patient stays in NICU or incubator after birth was also independently associated with lower communication, gross motor, fine motor, and problem-solving domains, after adjusting for LBW (*p*-values < 0.05). In addition, newborns that received resuscitation had − 0.43 (95% CI -0.83, − 0.03) and − 0.78 (95% CI: − 1.30, − 0.25) SD lower communication and fine-motor developmental scores, respectively. Antibiotic use, a proxy of neonatal infection, was associated with poorer fine motor (SMD: -0.32; 95% CI: − 0.6, − 0.05) and problem-solving (SMD: -0.28; 95% CI: − 0.5, − 0.07). In multivariate models, not initiating breastfeeding within the first hour after birth was associated with − 0.30 SD (95% CI: − 0.51, − 0.09) lower gross motor and − 0.21 SD (95% CI: − 0.37, − 0.06) lower personal-social domain scores as compared to infants who initiated breastfeeding within the first hour after birth.

**Table 3:** Association of neonatal care factors with child developmental outcomes

	N	Communication	*p*-value	Gross motor	*p*-value	Fine motor	*p*-value	Problem-solving	*p*-value	Personal-social	*p*-value
		Multivariate-adjusted standardized mean difference (95% CI)		Multivariate-adjusted standardized mean difference (95% CI)		Multivariate-adjusted standardized mean difference (95% CI)		Multivariate-adjusted standardized mean difference (95% CI)		Multivariate-adjusted standardized mean difference (95% CI)	
***Neonatal care factors***^***a***^											
Extended in-patient stay after birth^a^											
NICU or Incubator	251	− 0.21 (− 0.41, − 0.01)	**0.04**	− 0.43 (− 0.77, − 0.09)	**0.01**	−0.38 (− 0.65, − 0.11)	**0.006**	− 0.17 (− 0.42, 0.07)	0.17	−0.22 (− 0.41, − 0.03)	**0.02**
Nursery	274	− 0.01 (− 0.14, 0.11)	0.83	−0.02 (− 0.13, 0.09)	0.72	−0.02 (− 0.24, 0.19)	0.81	− 0.13 (− 0.29, 0.01)	0.08	0 (− 0.13, 0.14)	0.98
Routine Discharge	2984	Reference		Reference		Reference		Reference		Reference	
Need for resuscitation after birth^a^ (Reference: no)											
Yes	36	−0.43 (− 0.83, − 0.03)	**0.03**	− 0.56 (− 1.45, 0.32)	0.21	− 0.78 (− 1.3, − 0.25)	**0.004**	−0.14 (− 0.77, 0.47)	0.64	− 0.46 (− 0.95, 0.02)	0.06
Antibiotics after birth (neonatal infection)^b^ (Reference: no)											
Yes	191	−0.18 (− 0.43, 0.07)	0.16	− 0.34 (− 0.72, 0.04)	0.08	−0.32 (− 0.6, − 0.05)	**0.02**	−0.28 (− 0.5, − 0.07)	**0.01**	−0.19 (− 0.44, 0.05)	0.13
Breastfed within the first hour of life^c^ (Reference: yes)											
No	732	−0.1 (− 0.24, 0.03)	0.13	− 0.3 (− 0.51, − 0.09)	**0.005**	−0.16 (− 0.41, 0.08)	0.20	−0.06 (− 0.21, 0.08)	0.39	−0.21 (− 0.37, − 0.06)	**0.007**

^a^adjusted for age, sex, interviewer, maternal level of schooling, permanent income, ferrous sulfate, folic acid, smoking, drinking, low birth weight, gestational age and twin birth

^b^adjusted for age, sex, interviewer, maternal level of schooling, permanent income, ferrous sulfate, folic acid, smoking, drinking, low birth weight, twin birth, and extended in-patient hospital stay after birth

^c^adjusted for age, sex, interviewer, maternal level of schooling, permanent income, ferrous sulfate, folic acid, smoking, drinking, low birth weight, twin birth, antibiotics, and need for resuscitation (selected to avoid collinearity)

General linear models, adjusted for sample clustering

**Fig. 1:**
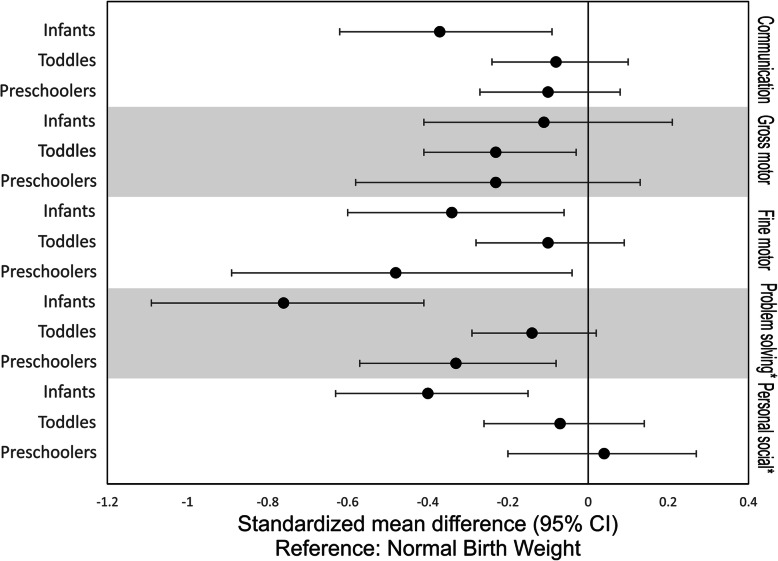
Forest plot of standardized mean difference of ASQ 3 scores (95% CI) in children born with low birth weight as compared to children born with normal weight. (* denotes statistically significant interaction)

The potential interaction of LBW with child age was also assessed (Table 4). In Fig. 1 is presented that the magnitude of the negative association of LBW with the problem-solving and personal-social domain scores was greater in magnitude for younger children (≤1 year) as compared to toddlers and preschoolers (*p*-values for the interaction < 0.05).

**Table 4:** Interaction between age and LBW as determinants of child development

	n	Communication	Gross motor	Fine motor	Problem-solving	Personal-social
		standardized mean difference (95% CI)	standardized mean difference (95% CI)	standardized mean difference (95% CI)	standardized mean difference (95% CI)	standardized mean difference (95% CI)
**Age**						
*In children < = 1 year (infants)* (reference: BW ≥ 2500 g)						
LBW	62	−0.37 (− 0.62, − 0.09)	−0.11 (− 0.41, 0.21)	−0.34 (− 0.6, − 0.06)	−0.76 (− 1.09, − 0.41)	−0.40 (− 0.63, − 0.15)
*In children > 1 year and < = 3 years (toddlers)* (reference: BW ≥ 2500 g)						
LBW	107	−0.08 (− 0.24, 0.1)	−0.23 (− 0.41, − 0.03)	−0.10 (− 0.20, 0.09)	−0.14 (− 0.29, 0.02)	−0.07 (− 0.26, 0.14)
*In children > 3 years (preschoolers)* (reference: BW ≥ 2500 g)						
LBW	109	−0.1 (− 0.27, 0.08)	−0.23 (− 0.58, 0.13)	−0.48 (− 0.89, − 0.04)	−0.33 (− 0.57, − 0.08)	0.04 (− 0.2, 0.27)
*p*-values for interaction by age		0.37	0.66	0.41	**0.04**	**0.04**

General linear models with interaction terms, adjusted for sample clustering

## Discussion

In this population-based survey conducted in Ceará, Brazil, we found that pregnancy and neonatal care factors were associated with development outcomes. In terms of pregnancy factors, children whose mothers did not receive folic acid supplementation during pregnancy had lower fine motor and problem-solving scores. LBW was associated with lower communication, fine-motor, and problem-solving scores. We also found the magnitude of the negative association for LBW was greater for infants as compared to older children for problem-solving and personal-social domain scores. In terms of neonatal care, need for resuscitation, antibiotic use by the newborn, and extended in-patient stay after birth was associated with lower development scores in selected domains. Initiation of breastfeeding within the first hour of life was associated with better gross motor and personal-social domains scores.

In terms of pregnancy factors, we found that not receiving folic acid supplementation during pregnancy was associated with large (> 0.5) deficits in the fine motor and problem-solving scores. Folate is essential in the formation of the neural tube [22]. It is also necessary for the production of RNA and DNA precursors, and low folate levels can result in abnormalities in cell proliferation, including for neuron production, and can contribute to DNA instability and chromosome breakage [23]. A recent observational study in China found that children whose mothers took folic acid supplements during pregnancy had significantly higher development quotient (DQ) at 1 month of age as compared to children whose mothers did not take folic acid [24]. Further, another study found that maternal serum folate concentration in late pregnancy was significantly associated with higher language development scores at 2 years of age [25]. As a result, our study adds to the growing observational evidence base that folic acid supplementation in pregnancy may improve development outcomes.

The prevalence of LBW and preterm birth found in this study is comparable with rates found in other Brazilian studies, and government data for Ceará in the year 2017 which recorded 8.2% LBW [26, 27]. There is a robust literature linking LBW to increased risk of suboptimal development outcomes, and some research has suggested that that language development may be the most affected domain [28, 29]. Many studies have identified that this association of LBW with development scores persists among school-age children and even into adulthood [10, 13]. It is estimated that approximately 25–50% of LBW infants have brain abnormalities associated with cognitive, behavioral, attentional, and socialization impairment [30, 31]. In this study, we found that LBW was a risk factor for suboptimal development for children up to 5 years of age. However, we found that the magnitude of the association tended to be greater for younger ages. This finding may be due to evidence of the developmental ‘catch up’ of the LBW children, which has been seen in other studies, in which LBW children aged 2 years of age and older would start to catch up with the rest of age-matched peers [32].

There are several mechanisms by which LBW may have an impact on child development. The process starts from the intrauterine formation of neuronal connections and extends after birth, implying a different growth of the corpus callosum, cerebral volume, and cortical thickness [31]. LBW may be the result of prematurity and intrauterine growth restriction. We found that LBW was associated with impaired development independently of gestational age, but some of its effects may follow a pathway close to that of prematurity. Premature infants can have conditions like periventricular leukomalacia and accompanying neuronal/axonal abnormalities (common, occurring in 50% or more of very LBW infants < 2000 g); severe germinal matrix-intraventricular hemorrhage, and post-hemorrhagic hydrocephalus, which may directly affect development outcomes [33]. Besides, infants may be LBW due to intrauterine growth restriction (IUGR), indicating constraints in fetal nutrition that may lead to suboptimal development [34]. The evidence on the relationship of small-for-gestational-age newborns with development outcomes is mixed [35]. Besides the direct biological constraints due to prematurity and SGA, LBW infants have an increased need for parental care that may contribute to maternal stress, depression, and other factors that can lead to poorer child development outcomes [36]. In addition to the mechanisms mentioned earlier, children born with LBW have less ability to concentrate and impaired personal-social personal skills, as also seen in this study. This compromised social competence can generate a vicious circle of worsening in the development of older children [37]. Interventions to reduce the risk of LBW may have significant effects on developmental outcomes and programs to support the growth and development of preterm and LBW infants.

Few studies have assessed the association between neonatal care factors and developmental outcomes in low-income settings. After adjusting for LBW, we found that extended in-patient stays in the NICU or incubator after birth was associated with impaired communication, gross motor, and fine motor development. We also found that the need for resuscitation and antibiotic use after birth were independently associated with lower scores in communication and problem-solving domains and problem-solving and personal-social domains, respectively. The need for resuscitation can be considered a proxy of hypoxia, which can lead to brain injury and developmental impairment [38]. Neonatal infections, including sepsis, may have long-term effects on child developmental outcomes [39]. As a result, stimulation interventions and educational programs may consider targeting children who require extended in-hospital stays, require resuscitation, or have neonatal infections due to their risk for suboptimal developmental outcomes later in life, regardless of the birth weight.

Finally, we also found that breastfeeding initiation within the first hour of life was associated with improved gross motor and personal-social development. Breastfeeding within the first hour of life has a protective effect on neonatal mortality and morbidity; however, there is little evidence of its impact on developmental outcomes [40, 41]. It has also been associated with increased bonding between the newborn and the mother through increased skin-to-skin contact, promoting continued breastfeeding, and maybe a protective factor in LBW children, and thus, it should be encouraged [42].

This study is one of the first evaluations of the association between LBW and children’s development in a pediatric sample with a broad age range in a developing country, with a state-wide representative sample. Nevertheless, this study has a few limitations. First, the study’s cross-sectional design does not allow the analysis of child development trajectories over time, nor directs the determination of causal associations and recall bias can impact information in older children. Second, we used the ASQ-3, a validated screening tool that allows child development evaluation in a large populational sample, but which is not a diagnostic tool for child developmental delay. Furthermore, while the study was designed to represent a pediatric population in the State of Ceará, our findings may not be generalizable to children in other contexts.

## Conclusions

We determined that pregnancy and neonatal factors were associated with child development in a population-based study in Ceara, Brazil. We found that lack of folic acid supplementation in pregnancy, LBW, newborn resuscitation, newborn receipt of antibiotics and extended in-patient stays were risk factors for suboptimal development outcomes in selected domains. As a result, these findings suggest these high-risk groups may benefit from supplemental interventions, such as LBW or infants requiring extended in-patient stay. However, it is important to note that integrated interventions to prevent adverse pregnancy and birth outcomes may also have positive effects on child development. Research on population-based interventions to improve child development in Brazil is warranted.

## Data Availability

The datasets used and/or analyzed during the current study are available from the corresponding author on reasonable request.

## Abbreviations

LBW: Low Birth Weight
SGA: Small for gestational age
PESMIC: *Pesquisa de Saúde Materno Infantil do Ceará*
IBGE: Brazilian Institute of Geography and Statistics
USDA: United States Department of Agriculture
ANC: Antenatal care
L.M.I.C.: Low- and middle-income countries
S.D.G.: Sustainable development goals
NICU: Neonatal Intensive Care Unit
